# Microbial keratitis in the age of resistance: unlocking the therapeutic potential of phage therapy

**DOI:** 10.1007/s00417-025-06961-z

**Published:** 2025-09-18

**Authors:** Rafwana Ibrahim, Pritam Kumar, Jesil Mathew Aranjani

**Affiliations:** Department of Pharmaceutical Biotechnology, Manipal College of Pharmaceutical Sciences, Manipal Academy of Higher Education, Manipal, 576104 India

**Keywords:** Microbial keratitis, Phage therapy, Antibiotic resistance, Biofilm, Novel drug delivery, Global health

## Abstract

Keratitis an inflammatory disorder of the corneal tissue, poses a significant threat to vision and, if left untreated, can even progress to irreversible blindness. Clinical manifestations of the disease include ocular redness, pain, photophobia, excessive tearing, and visual disturbances, with severe cases often leading to corneal ulceration, scarring, or perforation. The global prevalence of keratitis exhibits substantial geographical variability, largely influenced by access to healthcare, environmental factors, and behavioral risk determinants, most notably, the use of contact lenses. In recent years, microbial keratitis (MK) has shown a concerning rise in incidence, particularly among contact lens users, frequently attributed to improper lens hygiene and extended wear. Current therapy mainly depends on the intensive use of topical antimicrobial agents; however, the emergence of multidrug-resistant (MDR) pathogens and the protective nature of biofilms significantly compromise therapeutic efficacy and efficiency. These limitations pave the way to the urgent need for alternative strategies. Bacteriophage therapy, which was in use even before the development of antibiotics, regained interest as a precision-based antibacterial treatment that is capable of selectively targeting pathogenic bacteria, including MDR strains, without disrupting the native ocular microbiota or damaging host tissue. This review explores the different types of keratitis and pathogenesis, highlights the problems related to conventional therapies, and emphasizes the potential of phage therapy as a novel, adjunctive, or standalone intervention.

## Abstract (fr)

**Key messages:**

*****What is known***:**

- Microbial keratitis (MK) is a serious ocular infection that can lead to blindness, with rising prevalence among contact lens users.

- The effectiveness of conventional antimicrobial therapy is increasingly compromised by multidrug-resistant (MDR) pathogens and biofilm-associated protection.

*****What is new***:**

- Bacteriophage therapy offers a promising, targeted approach against MDR pathogens in microbial keratitis without disrupting the native ocular microbiota.
- Phages can penetrate biofilms and have the potential to act as adjunctive or standalone treatments where antibiotics fail.
- This review underscores phage therapy as a viable alternative and brings attention to its renewed relevance in ophthalmic infectious disease management.

## Introduction

Microbial keratitis (MK) is a severe, vision-threatening corneal infection that requires immediate and effective treatment in order to avert irreversible visual function and blindness [1]. It is predominantly caused by bacterial, fungal, viral, or protozoal pathogens, with the use of contact lens being a significant predisposing factor [2]. The global incidence varies extensively and ranges between 0.36 and 79.9 per 10,000 people per year, with a large proportion being related to the use of a contact lens, especially in areas with a high usage pattern [1]. South, Southeast, and East Asia have over 2 million annual cases, with the magnitude representing a considerable disease burden [3]. Contact lens use confers a particular risk for MK, with unhygienic use, extended-wear use, and overnight wear being well-defined risk determinants [4]. A global incidence based on epidemiological studies estimates that the incidence related to the use of a contact lens occurs in 2 to 20 per 10,000 users per year, making prevention and treatment a necessity [5]. Keratitis is a global health problem with appreciable variance in incidence based on geographical location, infrastructure, and risk determinants including the use of a contact lens. Global incidence varies between 2.5–799 per 100,000 people per year [1]. In the United States, annual incidence ranges between 11.0 and 27.6 per 100,000 people, with a trend increase in ulcerative keratitis, especially among the use of a contact lens population [2, 6]. On the other hand, India reports the disease at a much greater magnitude, with the incidence being as high as 113 per 100,000 people, especially among rural areas with a high activity pattern in the agricultural sectors with a high fungal keratitis incidence [7]. Nepal boasts one of the highest reported rates of incidence with as much as 799 per 100,000 person-years, particularly in a setting with unhygienic conditions and a lack of access to care facilities[8]. Incidence rates in nations with good health care, including Denmark, are lower in comparison. Herpes simplex keratitis alone contributes to around 1.5 million fresh instances each year, resulting in up to 40,000 fresh instances of blindness per year [9]. It takes a significant economic toll, making prevention and treatment essential, especially in regions with a high incidence.

Beyond just causing vision loss, microbial keratitis has major financial and medical costs. Hospitalization, extended antibiotic treatment, surgery, and lost productivity all have high financial expenses that put a significant burden on both individuals and healthcare systems [10, 11]. Severe cases may worsen resource utilization by developing corneal perforation and scarring, which would require corneal transplantation. Microbial keratitis patients'quality of life is severely impacted by their severe ocular pain, photophobia, profuse lacrimation, and visual abnormalities [12, 13]. Even with vigorous therapy, persistent corneal opacities or vision loss frequently continue, limiting daily activities and occupational performance. Rapid disease development calls for immediate management. In addition to physical morbidity, MK has profound psychosocial consequences, as affected individuals experience heightened anxiety, depression, and social limitations due to their condition [14]. The necessity for prolonged medical care and strict adherence to treatment regimens further compounds the psychological burden associated with the disease.

Managing microbial keratitis (MK) typically begins with a primary treatment regimen of antimicrobials, including intensive topical antibiotics, antifungals, or antivirals, depending on the underlying cause [15]. The use of corticosteroids to control inflammation is still a concern because they might worsen the infection and the risk of side effects [16]. Even with these strategies, there is a rise of multidrug-resistant (MDR) pathogens, particularly *Pseudomonas aeruginosa* and *Staphylococcus aureus*, which will further complicate treatment, especially in contact lens-associated cases [17]. The overuse of antibiotics is considered to be a major reason for the accelerated resistance, making standard therapies less effective. In addition, issues like poor drug penetration into the corneal stroma, frequent dosing, and drug-related toxicity further limit treatment success.

Bacteriophage therapy has emerged as a promising alternative to conventional antimicrobial treatment, offering a precise mechanism of bacterial eradication [18, 19]. Bacteriophages preferentially infect and eradicate bacterial pathogens, making them a precise, effective and specific replacement for MDR strains of bacteria [20]. Unlike broad-spectrum antibiotics, phage therapy preserves the commensal microbiota, reducing dysbiosis and subsequent infections [21, 22]. The replicating property of phages at the site of infection in the presence of a causative pathogen makes long-term antibacterial efficacy possible. Historically, phage therapy has shown encouraging results in treating antibiotic-resistant diseases like eye infections. Because studies have demonstrated reduced bacterial loads, improved corneal healing, and fewer side effects when used to treat infections caused by *P. aeruginosa* and *S. aureus*, phage mixes may be a helpful treatment for microbial keratitis [23, 24].

Phage treatment presents obstacles and constraints despite its encouraging potential. It is necessary to resolve bacterial resistance to phages, formulation stability problems, and regulatory barriers before broad clinical implementation. Integrating phage preparations into ordinary clinical practice is complicated by the requirement for customized preparations and the lack of established treatment procedures. However, stability, efficacy, and regulatory approval improvements are made possible by formulation technology, synthetic biology, and phage engineering developments. Phage therapy has the potential to completely transform the treatment of ocular infections as long as research into it is conducted [25]. Future research should concentrate on streamlining delivery systems, clarifying host-phage interactions, and developing strong clinical validation techniques to enable the smooth integration of phage-based treatments into standard ophthalmic practice.

This review provides a thorough overview of the various types of keratitis, focusing on the underlying pathophysiology, host immune responses, current therapeutic approaches, their limitations, and the emerging role of bacteriophage therapy as a novel treatment strategy.

### Microbial keratitis

Keratitis is a major inflammatory disease caused to the cornea, which may be associated with various infectious micro-organisms like amoeba, bacteria, viruses, and fungus and may also be caused by non-microbial factors like trauma, chemical exposure, ultraviolet exposure, etc. Keratitis is associated with intense pain and usually impaired eyesight, photophobia, red eye, and a gritty sensation [1, 2].

Contact lens wear is one of the major cause of microbial keratitis. Earlier before the introduction of contact lenses as an alternative to spectacles, the major risk factors that were associated included trauma and microbial growth during inflammation and infections [26]. After the introduction of contact lenses in the 1970 s, contact lens wear became one of the predisposing factors for microbial keratitis due to poor hygiene, extended wear, and contact lenses associated with ocular inflammations [27, 28]. Among these, the extended wear of contact lenses become the major risk factor for microbial keratitis. During contact lens wear, the chances for the microbes to enter the eye from the wearer’s lid margins, their fingers upon lens insertion (or removal), or via the contact lens, from the care solutions, or the storage case is high. As an additional source of contamination improper use of lenses and their disinfection/cleaning systems plays a major role [29].

Another important factor is the impact of contact lens wear on tear fluid, which protects the corneal epithelial cells against *Pseudomonas aeruginosa*. The tear fluid acts against the microorganisms by directly affecting them or by upregulation of the defensive capacity of the epithelial cells [30, 31]. A contact lens has the ability to alter tear biochemistry at the corneal surface when it comes too close to the cornea or reduce the tear mixing between the pre and post-lens tear compartment during blinking. The changes caused to ocular surface biochemistry could lead to various consequences including loss of direct or indirect defense against microbes.

### Types of keratitis

Keratitis can be caused by either infectious or noninfectious agents, leading to varying degrees of visual impairment and leads to blindness if left untreated.

**Fig. 1:**
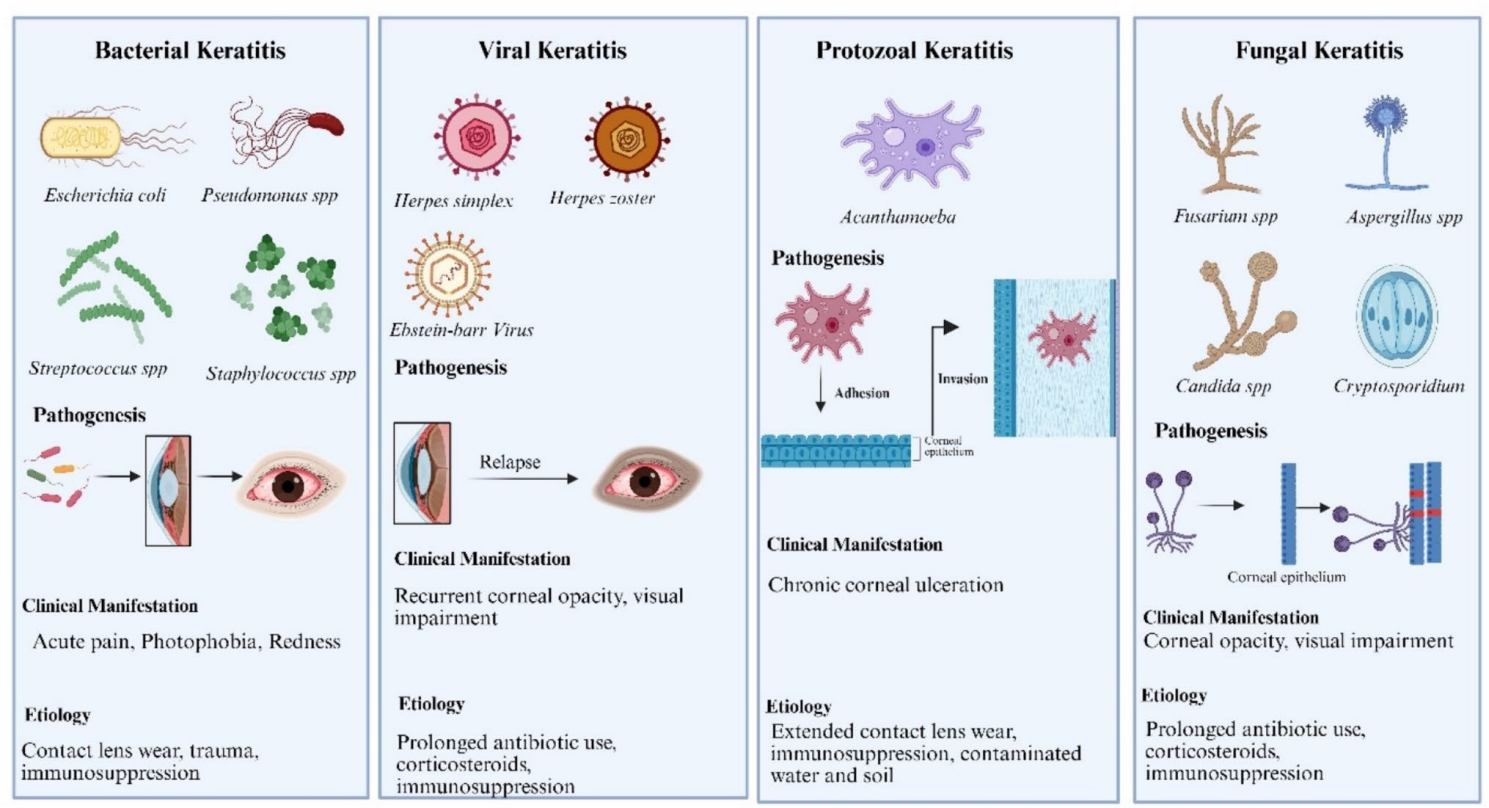
Types of microbial keratitis

Infectious keratitis is caused by microbial pathogens such as bacteria, viruses, fungi, and protozoa [32, 33]. It often presents symptoms such as ocular pain, redness, photophobia, and vision impairment. Bacterial keratitis is a rapidly progressing infection of the cornea that is commonly associated with trauma, contact lens wear, ocular surface disorders, and immunosuppression (Fig. 1). The most frequently implicated bacterial species include *Staphylococcus* spp.*, Pseudomonas aeruginosa, Streptococcus pneumoniae, Corynebacterium* spp.*, and Moraxella spp* [34, 35]. The bacterial keratitis emerges with the bacterial adherence to the corneal epithelium, secretion of extracellular enzymes and toxins, and subsequent immune activation, leading to stromal necrosis and ulceration [36]. Clinically, bacterial keratitis presents with severe pain, corneal edema, hypopyon formation, and progressive stromal infiltration [35]. The conventional therapy typically includes broad-spectrum antibiotics such as fluoroquinolones (moxifloxacin, gatifloxacin), aminoglycosides (gentamicin, tobramycin), and cephalosporins, with therapy adjusted on the basis of culture and susceptibility results [15].

Viral keratitis, predominantly caused by herpes simplex virus (HSV-1), is considered to be the major cause of corneal infections that lead to blindness worldwide. Other causative agents include varicella-zoster virus (VZV) and cytomegalovirus (CMV) [37, 38]. The mechanism of viral keratitis mainly follows a latency trend where the virus establishes latency in the trigeminal ganglion, which gets reactivated upon stress, immunosuppression, or trauma, leading to recurrent corneal infections [39]. The clinical manifestations of viral keratitis include epithelial keratitis (dendritic or geographic ulcers), stromal keratitis (necrotizing or nonnecrotizing inflammation), endotheliitis (disciform keratitis), and neurotrophic keratopathy due to corneal nerve damage. The treatment regimen mainly consists of antiviral agents such as acyclovir, valacyclovir, and ganciclovir, whereas corticosteroids are used in patients with stromal keratitis to modulate the inflammatory response [40].

A severe, sight-threatening inflammatory condition that is often associated with ocular trauma is fungal keratitis. The main reasons include the plant material, prolonged corticosteroid use, and contact lens wear. The common pathogens *Fusarium* spp.*, Beauveria bassiana, Aspergillus* spp.*, and Candida spp* are the predominant causes of fungal keratitis [41]. Disease pathogenesis involves deep stromal penetration by fungal hyphae, immune evasion, and chronic inflammation. Clinical features include corneal infiltrates with feathery borders, satellite lesions, hypopyons, and intense pain. Management strategies involve topical antifungals such as natamycin, voriconazole, or amphotericin B, with surgical interventions reserved for refractory cases [36, 42].

Among the microbial keratitis, well-known is Acanthamoeba keratitis, which is a rare but severe corneal infection that primarily affects contact lens users. The primary causative agent is *Acanthamoeba spp*., which adheres to the corneal epithelium then invades more profound tissues and induces chronic inflammation and nerve damage. Clinical symptoms include severe pain disproportionate to clinical findings, ring-shaped stromal infiltrates, and progressive corneal ulceration [43, 44]. Treatment involves prolonged administration of biguanides including polyhexamethylene biguanide and chlorhexidin in combination with diamidines like propamidine, hexamidine etc. [45].

Non-infectious keratitis arises from autoimmune diseases, trauma, or inflammatory conditions and is often associated with systemic immune dysregulation. Interstitial keratitis and the peripheral ulcerative keratitis are considered to be the common non-infectious keratitis [46, 47].

Interstitial keratitis is a chronic corneal stroma inflammatory disorder frequently linked to infectious or autoimmune etiologies. Common causes include syphilis caused by *Treponema pallidum*, viral infections, parasitic antigens, and systemic autoimmune disorders. Pathogenesis involves immune complex deposition, leading to non-suppurative stromal inflammation and vascularization. Clinically, interstitial keratitis presents with stromal haze, corneal thinning, and ghost vessels. Treatment involves systemic antibiotics in patients with syphilis and corticosteroids to control inflammation [48, 49].

In case of Peripheral ulcerative keratitis is an immune-mediated inflammatory condition characterized by peripheral corneal thinning and ulceration. It is commonly associated with systemic autoimmune diseases such as rheumatoid arthritis, granulomatosis with polyangiitis, and systemic lupus erythematosus. Pathogenesis involves immune complex deposition and inflammatory cytokine activation, leading to keratolysis and neovascularization. The clinical presentation of this disease includes crescentic peripheral stromal ulceration and limbal inflammation. Treatment typically involves immunosuppressive therapy, corticosteroids, and biologics such as rituximab [50, 51] (Table 1).

**Table 1:** Type of keratitis

Type	Causative agents	Risk factors	Pathophysiology	Clinical manifestations	Treatment
Bacterial Keratitis	*Staphylococcus spp., Pseudomonas aeruginosa, Streptococcus pneumoniae, Corynebacterium spp., Moraxella spp.*	Contact lens wear, trauma, ocular surface disorders, immunosuppression	Bacterial adherence, toxin production, immune activation, stromal necrosis	Corneal edema, hypopyon, stromal infiltration, severe pain	Fluoroquinolones, aminoglycosides, cephalosporins
Viral Keratitis	*Herpes simplex virus (HSV-1), Varicella-zoster virus (VZV), Cytomegalovirus (CMV)*	Stress, immunosuppression, and trauma	Latency in trigeminal ganglion, viral reactivation, corneal nerve damage	Dendritic ulcers, stromal inflammation, neurotrophic keratopathy	Acyclovir, valacyclovir, ganciclovir, corticosteroids
Fungal Keratitis	*Fusarium spp., Beauveria bassiana, Aspergillus spp., Candida spp.*	Ocular trauma, corticosteroid use, contact lens wear	Hyphal penetration, immune evasion, chronic inflammation	Feathery infiltrates, satellite lesions, intense pain, hypopyon	Natamycin, voriconazole, amphotericin B
Protozoal Keratitis	*Acanthamoeba spp.*	Contact lens contamination, water exposure	Adherence to cornea, deep tissue invasion, nerve damage	Severe pain, ring-shaped infiltrates, progressive ulceration	Polyhexamethylene biguanide, chlorhexidine, propamidine, hexamidine
Interstitial Keratitis	*Treponema pallidum*	Syphilis, viral infections, autoimmune disorders	Immune complex deposition, stromal inflammation, vascularization	Stromal haze, corneal thinning, ghost vessels	Antibiotics (if syphilitic), corticosteroids
Peripheral Ulcerative Keratitis (PUK)	Autoimmune-mediated	Rheumatoid arthritis, granulomatosis with polyangiitis, lupus	Immune complex deposition, cytokine activation, keratolysis	Peripheral stromal ulceration, limbal inflammation	Immunosuppressives, corticosteroids, and rituximab

### Pathophysiology of bacterial keratitis

Bacterial pathogens infiltrate the corneal epithelium and stroma to cause bacterial keratitis, a serious and potentially blinding corneal infection. Environmental risk factors, host immune responses, and microbial virulence factors interact intricately in the pathophysiology of bacterial keratitis [36, 52].

**Fig. 2:**
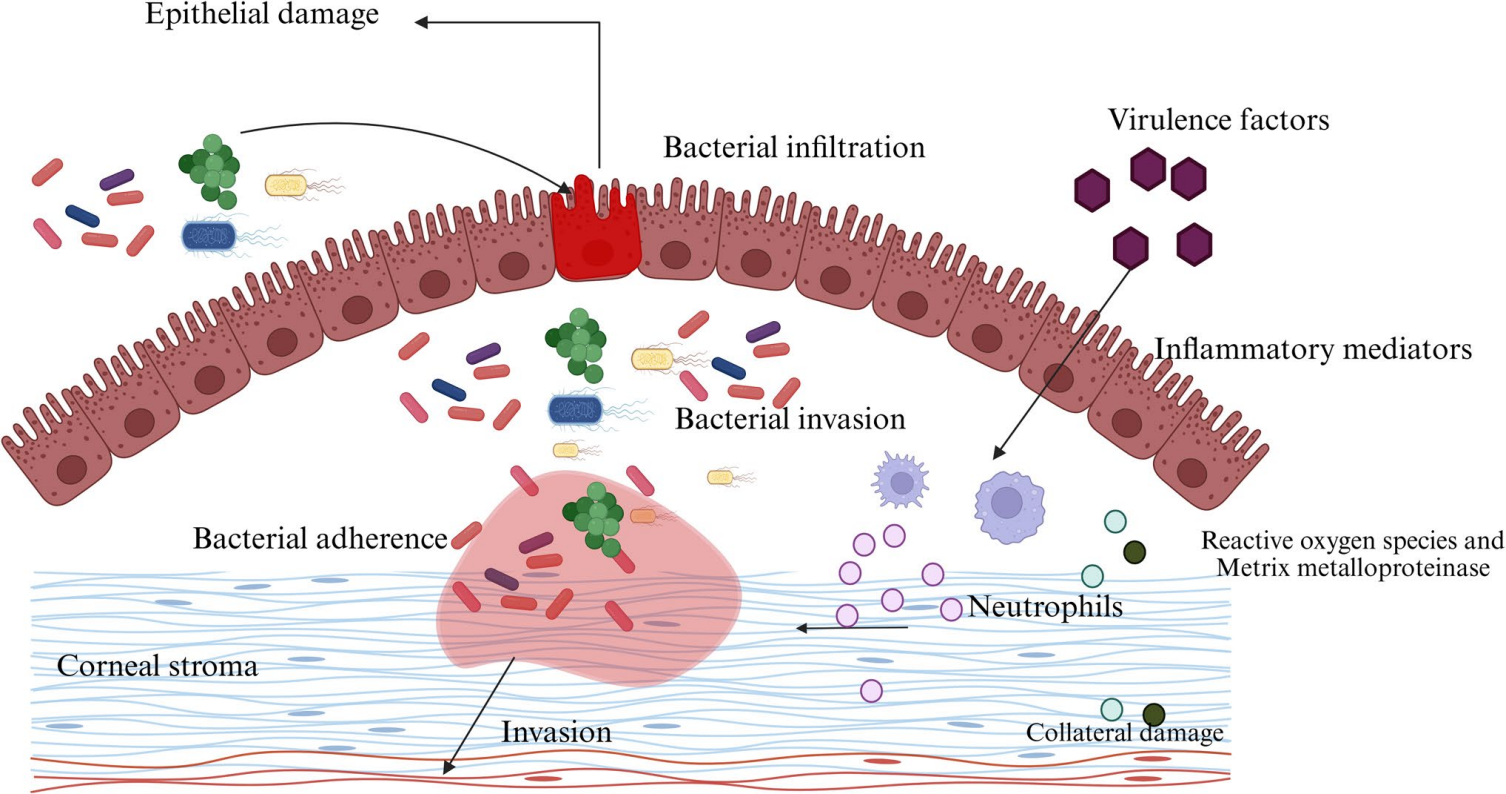
Mechanism of bacterial keratitis pathogenesis. The sequential events that occur during bacterial keratitis are depicted in the diagram. As an initial stage of microbial keratitis, bacteria adhere to the corneal epithelium, followed by damage and infiltration of the epithelium. Inflammatory mediators and bacterial virulence factors help and support the deeper penetration into the corneal stroma. Recruiting neutrophils and releasing reactive oxygen species and matrix metalloproteinases sets off an immune response that causes additional inflammation and collateral tissue damage

Corneal epithelial trauma, which can result from a number of underlying causes such as contact lens use, ocular surgery, or physical trauma, is the main way that infections spread. Opportunistic pathogens like *Pseudomonas aeruginosa, Staphylococcus aureus, and Streptococcus pneumoniae* penetrate the corneal tissue and start an inflammatory cascade when the corneal epithelial barrier is weakened for any of the reasons listed above [53]. After entering the cornea, bacteria use virulence factors like adhesins, toxins, and proteases to spread infection and elude the host's immune system. Different virulence factors contribute to bacterial keratitis in different ways. Bacterial attachment to corneal epithelial cells is made possible by adhesins, which facilitates tissue colonization [52–55]. For instance, *Staphylococcus aureus* makes fibronectin-binding proteins to bind to host tissues, while *Pseudomonas aeruginosa* uses pili and flagella for motility and adherence [56]. Bacteria release cytolytic toxins, such as hemolysins and exotoxins, after adhesion, which compromise the integrity of the corneal epithelium and encourage the spread of the bacteria. Collagenase, alkaline protease, and elastase are examples of proteolytic enzymes that aid in corneal deterioration, which results in ulceration and necrosis (Fig. 2).

The development of bacterial keratitis is also significantly influenced by the inflammatory reactions. When bacteria invade, corneal epithelial cells and resident immune cells use pattern recognition receptors (PRRs), like Toll-like receptors (TLRs), to identify pathogen-associated molecular patterns (PAMPs). Proinflammatory cytokines, such as interleukin (IL)−1β, tumor necrosis factor-alpha (TNF-α), and IL-6, are released when these PRRs are activated and draw neutrophils to the infection site. Although neutrophils are necessary for removing bacteria, excessive infiltration can cause tissue damage to bystanders because it releases matrix metalloproteinases (MMPs) and reactive oxygen species (ROS) [57, 58].

Bacterial replication and host immune responses worsen corneal destruction as the infection worsens. If severe bacterial keratitis is not treated, it can result in endophthalmitis, corneal perforation, and blindness. The bacterial load impacts the degree of corneal damage, host immune function and prompt antimicrobial therapy initiation. Developing targeted therapies to reduce infection and maintain corneal function requires understanding the molecular mechanisms underlying bacterial keratitis.

### Immune response in bacterial keratitis

The response to bacterial keratitis involves both innate immunity and adaptive immunity in a level of interaction that can be termed as intricate. The immune privileged tissues, such as the cornea, usually keep a balance between host defense mechanisms, inflammation, and control in order to preserve transparency and functionality. If the host equilibrium is disrupted by a pathogen fortifying the cornea, this leads to keratitis, unchecked inflammation, and progressive tissue destruction. The innate immune system serves as the first line of defense after bacteria invasion. Corneal epithelial cells act as frontline guards. They possess recognition receptors, such as Toll-like receptors (TLRs) and nucleotide oligomerization domain (NOD)-like receptors (NLRs), that contain pathogen-associated PAMPs [59, 60]. When activated, these receptors undergo intracellular signaling cascades, producing chemokines, antimicrobial peptides (AMP), and pro-inflammatory cytokines. Some of these AMPs include cathelicidins and defensins that compromise bacterial membrane integrity and directly execute a bactericidal effect [61]. Defensins who are anti-microbial are one of the major features of neutrophil infiltration equipped with enzymes which quickly respond to bacterial keratitis. Neutrophil attraction to the infection site is induced by pro inflammatory cytokines such as IL-8 released by the infected corneal epithelium. These defense cells apply a range of antibacterial methods, including neutrophil extracellular traps (NETs), degranulation, and phagocytosis. Even though uncontrolled neutrophil activity usually theraby causes border collateral tissue destruction from the uncontrollable secretion of reactive oxygen species (ROS) and proteolytic enzymes, and tissue damage to the cornea opacity and thinning, it does remarkably manage infection [62, 63].

At the same time, the adaptive part of the immune system also gets activated. Prior to moving to regional lymph nodes, dendritic cells in the cornea capture and process the bacterial antigens so these stem T cells can be presented to them. The primary expansion is the Th1-type immune response characterized by interferon-gamma (IFN-γ) secretion, which increases the macrophage’s bactericidal activity. By secreting anti-inflammatory cytokines such as interleukin-10 (IL-10) and transforming growth factor-beta (TGF-β), regulatory T cells (Tregs) help to restrain inflammation and, therefore, tissue damage, which strongly alters the balance of immune response [52, 64, 65]. Phagocytic cells are vital in getting rid of the bacteria; however, too much support, especially towards the cornea, can lead to serious damage with scars and loss of sight. In order to combat this, immunomodulatory drugs such as corticosteroids are frequently used in therapeutic approaches to reduce excessive inflammation. Biological agents specifically targeting inflammatory cytokines are being investigated in more sophisticated settings. Innovative immunotherapeutic platforms, such as delivery systems based on nanotechnology that combine antibacterial and anti-inflammatory agents, have also been the focus of recent research. Ultimately, these dual-purpose systems seek to maintain corneal clarity and visual function by eliminating infection and reducing immune-mediated tissue damage.

### Challenges in current therapeutic approaches for microbial keratitis

The treatment of microbial keratitis faces one of the greatest difficulties in contemporary medicine: the development of antimicrobial resistance (AMR). The increasing resistance of ocular pathogens has led to the marked reduction of efficacy of many first-line antimicrobial treatments, worsening clinical complexities. The existence of multidrug-resistant organisms caused by inappropriate antibiotic use overwards treatment possibilities [1, 66]. Most notably, pathogens such as *Pseudomonas aeruginosa* and *Staphylococcus aureus* increasingly resist commonly prescribed fluoroquinolones and aminoglycosides, loosening standard treatment reliability [67]. Fungal keratitis is challenging, with organisms developing resistance to vital antifungals such as natamycin and voriconazole. With few emerging antifungal drugs, this inadequacy intensifies the already dire need of novel therapeutic approaches [41, 68].

Even though they remain the foundation of treatment, topical aminomicrobials often have difficulty attaining therapeutic levels in the deep cornea structures. The ocular surface is critically protective but forms a very tight seal for permeation of drugs that can assist in treatment. This drawback tends to result from enhanced dosing schedules that can worsen adherence and hence treatment outcomes [69]. The situation is worse in fungal keratitis, where effective topical formulations are non-existent. Therefore, clinicians have to frequently rely on systemic antifungal treatment which is associated with the high risks of systemic toxicity and suboptimal concentration of the drug at the infection [41, 70]. More advanced drug delivery systems such as nanoparticles and liposomes have the potential to enhance ocular delivery of drugs although many of these technologies are still in developmental phases and not readily accessible in clinical settings.

Timely detection of the infectious agent is vital for effective treatment. But even this appears to be a principal limitation in the context of diagnosis. Conventional diagnostic methods such as Gram staining, KOH preparations, and microbial cultures are all suboptimal in sensitivity or erroneously slow for effective diagnosis, particularly for fungal or amoebic infections [71]. In contrast, molecular methods like PCR and next-generation sequencing have demonstrated greater accuracy and faster turnaround times [72, 73]. However, their broader implementation is limited by cost, infrastructure requirements, and the need for specialized personnel. Newer point-of-care diagnostic tools, such as biosensors and rapid multiplex assays, are being developed to bridge this gap and could transform the future of keratitis diagnostics by enabling quick and precise pathogen detection at the bedside or clinic.

The side effects related to antimicrobial and adjunctive therapies create significant problems. Chronic use of topical antibiotics, for instance, fluoroquinolones and aminoglycosides, has been linked with corneal toxicity, delayed epithelial healing, and in some cases, severe corneal melting all of which can profoundly impact visual outcomes [74–76]. Management of inflammation by corticosteroids is common but still remains controversial. Though they assist in reducing tissue damage due to overactive immune responses, their immunosuppressive action on local defenses can exacerbate infections, especially in keratitis herpetica and fungal keratitis. This paradox necessitates careful patient selection and dosing, which has led to consideration of other anti-inflammatory drugs that reduce inflammation without increasing microbial growth.

While a surgical approach is often deemed unfavorable, it is sometimes necessary. Therapeutic keratoplasty is done quite often to preserve the ocular tissue while restoring vision. Factors like advanced age, comorbid autoimmune diseases from long-term corticosteroid use, deep stromal involvement, large epithelial defects, and age may exacerbate poor outcomes [77, 78]. The development of bioengineered corneal substitutes and regenerative tissue technologies offers a path forward, potentially reducing the reliance on human donor tissue and improving surgical success rates.

Given the growing challenges AMR poses, along with diagnostic and therapeutic limitations, there is a critical need for innovative approaches to managing microbial keratitis. Emerging therapies offer promising solutions, ranging from antimicrobial peptides and biofilm-disrupting compounds to nanoparticle-based delivery systems and personalized medicine. Ongoing research must now focus on translating these advances into routine clinical use, ensuring they are both effective and accessible, with the ultimate goal of reducing the global impact of vision-threatening corneal infections [79, 80].

### Phage therapy: an alternative to treat microbial keratitis

Phage therapy has once again surfaced as an effective solution for dealing with infections that do not respond to conventional antimicrobial therapy. Bacteriophages, for instance, are viruses that target peculiar bacteria and can destroy them, something that conventional antibiotics cannot do precisely. Phages can destroy harmful bacteria by latching on to their specific receptors without disrupting the surrounding microbial community [81, 82]. This accuracy is of profound importance and at the same time makes phage therapy stand out when tackling issues associated with drug-resistant bacteria and chronic infections such as encountered in microbial keratitis where other options fail.

As already discussed, Microbial keratitis is a vision threatening inflammation of the eye caused by such bacteria as *Pseudomonas aeruginosa, Staphylococcus aureus*, and *Escherichia coli*. There is a high scope and platform to use phage therapy due to the increasing strength of these organisms to commonly used antibiotics which include fluoroquinolones and aminoglycosides. Furthermore, these pathogens increasing ability to form biofilms, or protective passes that shield and guard them from immune responses and potentially dangerous drugs adds to the complexity.

Unlike conventional therapies, phages have inherent enzymes that dissolve biofilm architecture enabling more effective bacterial infection control and improved infection control. Their replication at the infection site further enhances their therapeutic prospects [83, 84].

Research activities have already started to harness this promise. The phages designed to combat *P. aeruginosa* have proved to possess remarkable antibacterial potency, even against multi-drug resistant strains [23]. Laboratory and in vivo experiments have demonstrated that eye infections treated with phages resolve markedly more quickly, along with less inflammation and better healing of the tissues. Likewise, phages designed against methicillin-resistant *Staphylococcus aureus* (MRSA) have shown promising responses, including some activity against strains unresponsive to vancomycin. One of the strategies with increasing support is phage"cocktails"containing multiple phages directed toward different bacterial receptors, thereby broadening overall efficacy and reducing chances of resistance.

The data from preclinical models is very encouraging. In the rabbit model of *P. aeruginosa* keratitis, topical application of phages achieved comparable results to antibiotics in bacterial burden and corneal inflammation reduction [24]. Another study reported similar *S. aureus* eye infection treatment, including corneal clarity improvement and inflammatory marker reduction [85]. In spite of the lack of clinical trials, some patients undergoing phage therapy after failing conventional treatment showed promising results. One striking example is a drug refractory *P. aeruginosa* keratitis patient who fully recovered following topical and intracorneal phage therapy [86].

Even with these promising results waiting to be validated in clinical trials, there are still obstacles to overcome for phage therapy to be routinely adopted in ophthalmology practices. Phage specificity poses one of the greatest challenges phages are incredibly selective and finding the right one for the right infection is critical. This becomes a challenge if rapid identification of bacterial infection is not accessible [87, 88]. Construction of extensive, curated phage libraries will need to be accompanied with faster diagnostic test results to address the barrier.

Another concern is the potential for bacteria to develop resistance to phages, although this tends to occur less frequently than with antibiotics. Bacteria can evade phage attack through several mechanisms, such as changing surface receptors or activating innate defense systems like CRISPR–Cas [89]. To counter this, scientists are exploring engineered phages with enhanced capabilities and are looking at combining phage therapy with low-dose antibiotics to exploit their synergistic effects.

The immune system’s reaction to phage treatment is also worth noting. While systemic immune responses might interfere with intravenous or intramuscular phage use, topical application on the eye is expected to trigger minimal systemic effects. Still, long-term safety studies are needed, especially when repeated treatments are required.

The challenges posed by regulations and manufacturing practices are particularly different. Approving and standardizing protocols for phages, unlike with chemical drugs, is challenging because phages are biological entities that change over time. There is a lack of compassionate use frameworks for phage therapy within the FDA and EMA, but there is increasing interest in these therapies. As with all biopharmaceuticals, strict quality control, purification, stability, and control over the processes of phage production must be established in order to safely and consistently curtail variability. New formulation methods including encapsulation and freeze-drying are under research in order to improve phage stability and delivery to the eye.

More clinical research focused on eye infections is required in the coming years to fully optimize the usage of phages. Advanced delivery systems such as phage-loaded nanoparticles, bioadhesive gels, and even phage-impregnated contact lenses are some other avenues where research is needed along with optimizing dosing and evaluating long-term safety. Blending phage therapy with traditional antibiotics is also useful as other studies suggest using them at sub-therapeutic levels to enhance phage activity and inhibit bacterial resistance.

In summary, while challenges remain, the case for phage therapy in treating stubborn eye infections steadily grows stronger. With continued research, technological improvements, and regulatory support, this approach could offer a much-needed lifeline in the fight against resistant infections that threaten vision.

## Conclusion

Microbial keratitis is a significant public health issue, with severe cases frequently leading to decreased vision, monocular blindness, or even loss of an eye if not promptly treated and effectively. Microbial keratitis is most pronounced in countries as South and Southeast Asia and Africa, responsible for approximately 1.5–2 million new cases of monocular blindness annually. It constitutes a high rate of total corneal opacity and vision loss worldwide. The disease exists across all levels of severity [1, 90, 91]. It is affected by several factors, such as climate, economic level, accessibility to health care systems, and the widespread use of contact lenses. These geographical and demographic differences emphasis the need for targeted and precise prevention and management techniques, especially for high-risk individuals such as contact lens wearers, patients with ocular trauma, and the immunocompromised [91].

Conventional treatment of microbial keratitis is primarily driven by intense topical antibiotics or antifungal drugs, commonly in combination. With the increasing frequency of multidrug-resistant bacterial pathogens, particularly *Pseudomonas aeruginosa* and *Staphylococcus aureus*, combined with these pathogens'ability to form biofilms on the corneal surface or contact lenses, treatment regimens have become far more difficult. Biofilms form a physical and metabolic barrier that lowers the penetration and activity of antimicrobial agents, resulting in cases frequently recalcitrant to standard therapeutic approaches [91–93]. This has heightened the clinical demand for new and more effective therapy.

Bacteriophage therapy has re-emerged as a potential adjunct or alternative to bacterial keratitis treatment. Phages possess a particular lytic activity against their host bacteria, including MDR bacteria, and the potential for effective pathogen targeting in antibiotic-refractory cases. Preclinical studies in murine models have shown that topically administered phages are capable of potently decreasing corneal bacterial load and inflammation in MDR *Pseudomonas* and *Staphylococcus* species infection, and report some to be equal to or superior to those of standard antibiotic therapy [24, 94, 95]. These results strengthen phages'specificity and lytic activity against ocular pathogens in otherwise recalcitrant cases.

While these promising results are encouraging, most of the current evidence is still at the preclinical level. Clinical experience, while limited, is slowly building, as attested by case reports and small case series demonstrating compassionate use of topical phage products in patients suffering from drug-resistant or vision-threatening bacterial keratitis [86, 96]. Outcomes in such patients have demonstrated microbiological eradication and clinical healing, a proof of concept for prolonged use; however, randomized controlled trials in human subjects have not yet been performed. Advances such as phage-embedded hydrogels, in-situ forming eye drops, and other sustained-release drug delivery systems are on the horizon, and these are designed to improve phage stability and therapeutic residence on the ocular surface, thus circumventing some of the practical limitations of phage-based therapy for ocular disease [97].

In summary, while phage therapy is promising alternative for combating drug resistance and biofilm-associated keratitis, there is an urgent need for extensive further research. This includes conducting well-designed clinical trials to establish efficacy and safety in humans, optimizing delivery systems, and developing regulatory frameworks to support wider clinical integration. With continued advancements, phage therapy could offer a viable and targeted approach for difficult-to-treat resistant microbial keratitis patients.

In anticipation of future directions, concerted efforts will be required to translate the encouraging findings seen in preclinical and compassionate-use trials of phage therapy for keratitis into widespread clinical use. Principal goals are to initiate large-scale, randomized controlled trials to evaluate the safety, efficacy, and ideal dosing regimens for phage therapeutics, as monotherapy and in combination with standard antimicrobial drugs, against various pathogens and patient populations [98]. Developing well-curated phage libraries that are Good Manufacturing Practice (GMP) compliant will facilitate the rapid and tailored selection of phages that match clinical isolates, thereby circumventing the limitation imposed by narrow host range and resistance development [99].

There is a need for more technological development in drug delivery. Development of novel advanced forms, such as phage-embedded hydrogels, nanoparticles, and controlled-release systems, can increase phage stability, ocular surface residence time, and therapeutic effect. Pharmacokinetics, local residence, and tissue penetration experiments will be important to optimize these new delivery systems [86].

In parallel, further work should be carried out on synergizing phage therapy with existing antibiotics or anti-biofilm drugs to enhance antimicrobial activity and reduce the risk of phage resistance, and conventional drugs should be considered. Monitoring for safety and immunogenicity reactions, particularly during extensive or repeated therapy, will be required, as will the development of rigorous quality controls and regulatory standards for producing and using phage in the clinic [100].

International collaboration between investigators, clinicians, regulatory agencies, and industry partners will be required to meet these challenges and accelerate the clinical application of phage therapy. With these focused future directions, the field can optimize the potential of precision phage therapy to optimally enhance patient outcomes for keratitis and other recalcitrant ocular infections.
